# Humanized monoclonal antibody armanezumab specific to N-terminus of pathological tau: characterization and therapeutic potency

**DOI:** 10.1186/s13024-017-0172-1

**Published:** 2017-05-05

**Authors:** Michael G. Agadjanyan, Karen Zagorski, Irina Petrushina, Hayk Davtyan, Konstantin Kazarian, Maxim Antonenko, Joy Davis, Charles Bon, Mathew Blurton-Jones, David H. Cribbs, Anahit Ghochikyan

**Affiliations:** 1 Department of Molecular Immunology, Institute for Molecular Medicine, 16371 Gothard St, Huntington Beach, CA 92647 USA; 2 The Institute for Memory Impairments and Neurological Disorders, University of California, Irvine, Irvine, CA 92697 USA; 3 Sue and Bill Gross Stem Cell Research Center, University of California, Irvine, Irvine, CA 92697 USA; 4 Biostudy Solutions LLC, Wilmington, NC USA; 5 Department of Pharmaceutical Sciences, University of Nebraska Medical Center, Omaha, NE 68198-6025 USA

**Keywords:** Alzheimer’s disease, Tauopathy, Phosphatase activation domain, Immunotherapy, Monoclonal antibody, Humanization, Therapeutic efficacy

## Abstract

**Background:**

The experience from clinical trials indicates that anti-Aβ immunotherapy could be effective in early/pre-clinical stages of AD, whereas at the late stages promoting the clearing of Aβ alone may be insufficient to halt the disease progression. At the same time, pathological tau correlates much better with the degree of dementia than Aβ deposition. Therefore, targeting pathological tau may provide a more promising approach for the treatment of advanced stages of AD. Recent data demonstrates that the N-terminal region of tau spanning aa 2–18 termed “phosphatase activation domain” that is normally hidden in the native protein in ‘paperclip’-like conformation, becomes exposed in pathological tau and plays an essential role in the inhibition of fast axonal transport and in aggregation of tau. Hence, we hypothesized that anti-Tau_2–18_ monoclonal antibodies (mAb) may recognize pathological, but not normal tau at very early stages of tauopathy and prevent or decrease the aggregation of this molecule.

**Methods:**

Mouse mAbs were generated using standard hybridoma methodology. CDR grafting was used for humanization of mouse mAb. Humanized mAb (Armanezumab) was characterized and tested *in vitro*/*ex vivo*/*in vivo* using biochemical and immunological methods (HPLC, Biacore, ELISA, IHC, FRET, etc.). Stable DG44 cell line expressing Armanezumab was generated by clone selection with increased concentrations of methotrexate (MTX).

**Results:**

A panel of mouse mAbs was generated, clone 1C9 was selected based on binding to pathological human tau with high affinity and humanized. Fine epitope mapping revealed conservation of the epitope of human tau recognized by the parent murine mAb and Armanezumab. Importantly, Armanezumab (i) bound to tau with high affinity as determined by Biacore; (ii) bound pathological tau in brains from AD, FTD and Pick’s disease cases; (iii) inhibited seeding effect of aggregated tau from brain lysate of P301S Tg mice; (iv) inhibited cytotoxic effect of tau oligomers; (v) reduced total tau (HT7) and AT100, PHF1, AT8, AT180, p212, p214-positive tau species in brains of tau transgenic mice after intracranial injection. A stable CHO cell line producing >1.5 g/l humanized mAb, Armanezumab was generated.

**Conclusion:**

These findings suggest that Armanezumab could be therapeutic in clinical studies for treatment of AD.

## Background

Alzheimer’s disease (AD) is the most prevalent form of dementia worldwide. Compared to other deadly diseases, Alzheimer’s is the only disease that cannot yet be prevented, cured or slowed down. While death rates for other major diseases, such as heart diseases, cancer, AIDS etc., have declined, death rates from Alzheimer’s disease have risen by 71% since 2000 (www.alz.org). Since the “amyloid cascade hypothesis” was proposed, a primary focus of therapeutic approaches for AD have involved reducing amyloid-β (Aβ) levels in the brain [1, 2]. Anti-Aβ immunotherapy is considered as one of the most promising AD treatments under investigation, as it is currently being tested in many clinical trials [3–7]. Unfortunately, none of the attempts reported to date have shown positive clinical outcome in pivotal phase 3 trials. However, lessons learned from these trials indicate that, primarily, to be effective, anti-Aβ immunotherapy should be initiated before cognitive decline and severe pathological changes have occurred. Secondly, clearing Aβ at the late stages may be insufficient to halt the progression of AD. According to multiple reports, tau pathology correlates much better with the degree of dementia than Aβ plaque burden [8–14]. Targeting tau is now considered a promising approach for treatment of advanced AD stages. Additionally, tau is a common pathological marker for several neurodegenerative disorders collectively referred to as tauopathies, e.g. frontotemporal dementia with parkinsonism linked to chromosome 17 (FTDP-17) and Pick’s disease, corticobasal degeneration, progressive supranuclear palsy (PSP), amyotrophic lateral sclerosis (ALS), guam parkinsonism dementia complex, and dementia pugilistica. Therefore, development of safe and effective immunotherapy targeting pathological tau may become universal for the treatment of many diseases.

In this study, we generated and characterized monoclonal humanized version of a select mouse antibodies targeting a non-phospho-epitope located in N-terminal region of tau spanning aa 2–18 (tau_2-18_, known as PAD, phosphatase activation domain). This domain is normally hidden in a paperclip-like conformation of native protein, but becomes exposed in aggregated pathological tau [15]. It has been shown that exposed PAD plays an important role in the inhibition of anterograde fast axonal transport (FAT) as well as in polymerization of tau [16–21]. Immunohistochemical studies of human postmortem tissues and immunoreactivity with AD brain extracts with PAD specific antibodies (TNT-1) demonstrated that exposure of N-terminal region of Tau (TNT-1 immunoreactivity) is an early event in AD that is becoming more and more revealed in the severe stages of AD [16, 20–22]. Importantly, it was shown that appoptosin-mediated caspase-3 activation observed in several chronic neurodegenerative diseases including PSP, AD, FTD-T,HD, and PD, leads to tau cleavage at D421 [23–25]. Cleaved c-Tau is more prone to form aggregates/fibrils [25–29], associates with both early and late markers of NFTs in AD and is correlated with cognitive decline [29], strongly supporting the strategy aimed to target N-terminus of Tau exposed in caspase-cleaved c-Tau. Recently Bright et al. demonstrated the presence of extracellular N-terminal tau fragments secreted by iPSC cortical neurons from AD patients. Based on *in vitro* data they suggested that secreted extracellular Tau negatively impacts the neurons by inducing their hyperactivity and may elevate Aβ production in AD brain [30]. It is assumed that neutralization of these species can potentially slow the clinical progression of dementia.

Based on above mentioned data we hypothesized that antibodies generated against tau_2–18_ epitope may recognize pathological, but not normal tau at the early stages of tauopathy, and prevent/decrease the polymerization of this molecule.

We report the discovery of mouse mAbs targeting N-terminus of Tau leading to generation and characterization of a humanized anti-tau antibody, and culminating with development of a CHO cell line producing >1.5 g/l of this therapeutically potent Mab. Humanized antibody was termed Armanezumab according to *“Programme on International Nonproprietary Names (INN), Quality Assurance and Safety: Medicines, Essential Medicines and Pharmaceutical Policies (EMP)”* of WHO. Pending safety and therapeutic efficacy assessment, Armanezumab could be a candidate for clinical trials in mild to moderate AD patients.

## Results

### Generation of the precursor mouse antibody

A panel of monoclonal antibodies (mAb) were generated after immunization of mice with vaccine targeting epitope tau_2–18_ based on our proprietary MultiTEP platform (AV-1980R), which is highly immunogenic in mice, rabbits and monkeys [31–34]. Mice immunized with AV-1980R formulated with Quil-A adjuvant generated high titers of anti-tau antibodies that recognized not only tau_2–18_ peptide, but also full length human 4R/0N tau. Splenocytes from immunized mice were used for generation of hybridomas secreting monoclonal antibodies. After screening of 28 hybridoma clones for their ability to bind with full length tau and pathological tau by IHC to brains of patients with severe AD cases, clone 1C9 was selected for further characterization and humanization.

**Fig. 1:**
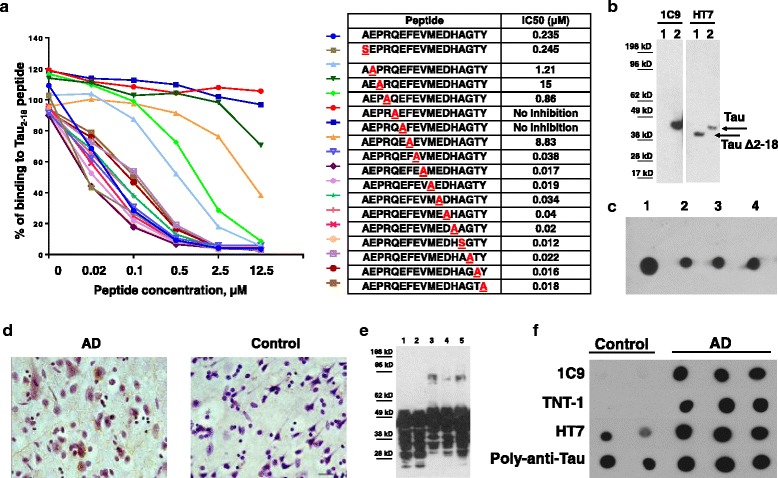
Characterization of mouse 1C9 anti-tau_2–18_ monoclonal antibody. **a** Competition ELISA using peptides with Alanine substitution showed that 1C9 recognized epitope PRQEF comprising 4–8 amino acids of tau_2–18_ peptide. The half maximal inhibitory concentration (IC_50_) for each peptide is shown in Table. **b** 1C9 recognized full-length tau but not tau that lacks 2–18 domain in Western Blot. Lane 1- tauΔ2-18, lane 2-full-length tau. **c** 1C9 bound monomeric (spot 1), oligomeric (spot 2: cross-linked; spot 3: non-cross-linked) and fibrillar (spot 4) forms of recombinant tau protein in dot blot. **d** Anti-tau_2–18_ mAb 1C9 bound to neuropil threads and neurofibrillary tangles in AD brains (Braak stage VI-C). No binding was observed with non-AD brain (Braak stage 0). Original magnification 40X, scale bar = 20 μm. **e** 1C9 bound different species of tau protein in brain homogenates from both AD cases and control subjects in denaturing conditions (lane 1: control 1; lane 2-control 2; lane 3-AD1; lane 4-AD2; lane 5-AD3) in Western Blot. **f** In non-denaturing conditions in Dot Blots 1C9 as well as commercial TNT-1 Ab specific to N-terminus of Tau selectively bound to soluble tau in AD brains but not in controls. Of note, HT7 and rabbit anti-tau-polyclonal Ab recognizing total tau, had bound tau in both control and AD brains

Fine epitope mapping of 1C9 antibody by alanine scanning demonstrated that substitution of residues 4–8 at the N-terminus of Tau to alanine effects the ability of antibody to bind to the peptide tau_2–18_, mapping the 1C9 epitope to PRQEF. Substitutions 6Q/A and 7E/A completely abrogated the competing ability of the peptides in competitive ELISA, showing that 6Q/7E are the most essential amino acids for binding to the antibody (Fig. 1a). Interestingly, substitutions of amino acids 9–18 slightly improved the binding ability of peptides, perhaps by promoting better exposure of the epitope by changing the structure of peptide. Specificity testing showed that this novel antibody recognized full-length recombinant tau, but not tau that lacks tau_2–18_ domain in western blot (Fig. 1b). 1C9 mAb bound different forms of recombinant tau: monomeric, oligomeric and fibrillar tau in dot blot assay (Fig. 1c). More importantly, 1C9 mAb recognized pathological tau (both neuropil threads and NFT) in the fixed brain sections from AD cases (Fig. 1d). Of note, 1C9 did not bind to the brain sections from a non-AD subject. In denaturing conditions of western blot 1C9 bound different forms of tau in homogenates of postmortem AD and control brains (Fig. 1e) showing a typical pattern as in case of HT7 antibodies recognizing total tau (see Fig. 3b). In contrast, in non-denaturing dot blot assay 1C9 selectively bound to soluble fraction of postmortem AD brain extracts (Fig. 1f) indicating that PAD is more exposed and accessible in the AD brains as opposed to controls.

### Generation of humanized antibody

**Fig. 2:**
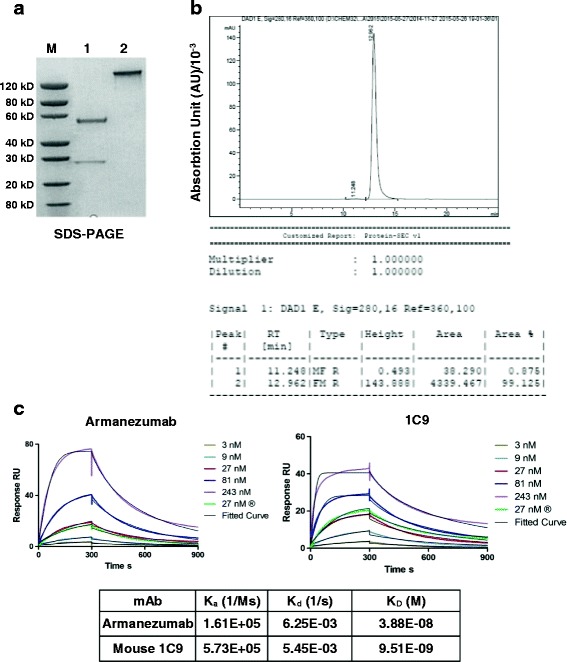
**a** SDS-PAGE analyses of purified antibody, Lane 1: Armanezumab, reducing conditions, 2.00 μg; Lane 2: Armanezumab, non-reducing conditions, 2.00 μg. **b** Armanezumab purified from CHO cells supernatant had a 99% purity measured by HPLC. **c** Characterization of Armanezumab and parental mouse 1C9 mAb by Surface Plasmon Resonance (SPR). SPR sensorgrams showing the binding of human tau (0 N/4R isoform) with each of immobilized antibody. Tau protein was run with various concentrations (3, 9, 27, 81, 243 nM), curves and fitted curve are shown in the corresponding color. Table shows the association rate constant (K_a_), dissociation rate constant (K_d_), and binding constant (K_D_) of antibodies with human tau. Biacore T200 evaluation software, version 1.0 was used to calculate K_a_ and K_d_ using 1:1 fitting model. Ms, millisecond; M, molar; s, second

Humanization of mouse antibody is necessary to minimize immunogenicity when the antibody is administered to humans, while retaining specificity and affinity of the parental non-human antibody. We used CDR grafting technology replacing CDR loops in the human mAb with mouse CDR loops and selected variable-region framework residues (as described in Materials and Methods). The reshaped, humanized antibody only retains essential binding elements from the murine antibody (5–10% of total sequence) and is predicted to generate only minimal immune responses in passively vaccinated subjects compared to chimeric human-mouse antibody (with replaced murine constant regions by human constant regions), which bears around 30% of original murine sequence [35]. The phage clone-producing antibody with the highest tau binding affinity was selected for expression as full antibody. Fc fragment of heavy chain of 1C9 was completely replaced with human IgG1 Fc fragment in the humanized version and the expressed humanized antibody, designated Armanezumab was purified for further analyses. Armanezumab was examined in SDS-PAGE (Fig. 2a) and HPLC (Fig. 2b), the latter analyses showed that its purity reached 99%.

**Fig. 3:**
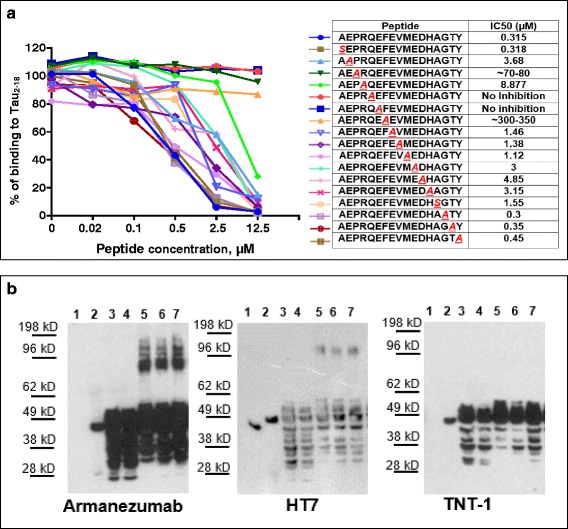
**a** “Alanine scanning” showed that CDR grafting did not affect the epitope specificity. Armanezumab recognized epitope PRQEF comprising 4–8 amino acids of tau_2–18_ peptide. Inhibition of binding of Armanezumab to Tau_2–18_ by peptides with alanine substitution in competition ELISA. The half maximal inhibitory concentration (IC_50_) for each peptide is shown in Table. **b** Armanezumab recognized (i) full-length recombinant tau protein, but not tau that lacks aa 2–18 (ΔTau2-18), lanes 1–2 and (ii) aggregated forms of tau in brain homogenates from AD cases (Braak stage VI), lanes 3–7. Lane 1: ΔTau2-18; lane 2: Full-length tau; lane 3: control brain 1; lane 4: control brain 2; lane 5: AD brain 1; lane 6: AD brain 2; lane 7: AD brain 3. HT7 recognizing total tau and TNT-1 specific to N-terminus of tau were used as positive controls

It is known that CDR grafting may alter the antibody’s binding capacity to the target antigen, so we compared binding affinity of Armanezumab with parental 1C9 mouse mAb measuring the equilibrium dissociation constant (K_D_) of both antibodies for binding to recombinant tau by SPR. As shown in Fig. 2c, the affinity of binding of humanized Ab is only slightly lower than mouse parental 1C9 mAb: K_D_ (M) of Armanezumab is 3.88E-08, while K_D_ (M) of 1C9 is 9.51E-09 (Fig. 2c). This difference between the affinities of binding of 1C9 mAb and Armanezumab is within the range observed for humanized antibodies compared with their parent mouse version. Fine epitope mapping of Armanezumab also showed that humanization process did not critically change the specificity of the antibody, except that IC50 for peptides with substitutions 4P/A and 8 F/A was higher indicating the importance of these amino acids for binding with Armanezumab (Fig. 3a).

### Functional characterization of Armanezumab

The functionality of Armanezumab was investigated using different approaches.

#### Binding of Armanezumab to pathological tau

**Fig. 4:**
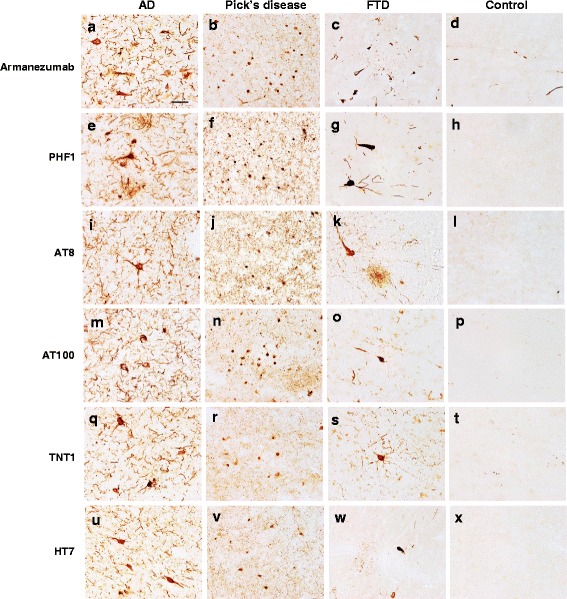
Armanezumab bound to pathological tau in brain tissues from inferior parietal gyrus of AD (**a**), midfrontal cortices of both Pick’s Disease (**b**) and Frontotemporal Dementia (**c**), while no binding was observed in the inferior parietal gyrus of non-AD brain (**d**). Adjacent brain sections stained with other antibodies against pathological tau such as PHF1 (**e**-**g**), AT8 (**i**-**k**), AT100 (**m**-**o**), N-terminal tau TNT1 (**q**-**s**), as well as HT7 anti-total tau (**u**-**w**), showed similar patterns of pathological profiles in perikarya and neuritic processes, while no binding was observed in the adjacent sections from the control brain (**d**, **h**, **l**, **p**, **t**, **x**). Original magnification 60X, scale bar =20 um

Our data showed that like 1C9, Armanezumab bound to full-length recombinant tau, monomeric and aggregated forms of tau in AD brain extracts and did not recognize recombinant tauΔ2-18 (Fig. 3b). It labels the similar pattern of tau protein as HT7 recognizing total tau. In contrast to commercial PAD-specific antibody TNT-1, Armanezumab bound also the aggregated forms of tau in brain extracts from AD patients. Importantly, Armanezumab recognized pathological tau in cortices not only from AD cases but also from Frontotemporal Dementia and Pick’s Disease, similarly to other antibodies specific to pathological or total tau, such as PHF1, AT8, AT100, HT7 and TNT-1. No binding was detected in control cortices from non-demented subjects (Fig. 4).

#### Armanezumab Inhibits seeding activity of aggregated tau

To assess a relevant therapeutic activity of Armanezumab we used cellular model that assays the seeding activity of pathological tau [36]. Previously it was demonstrated that fluorescence resonance energy transfer (FRET) assay could be used to track the aggregation of aggregation-prone tau containing P301S mutation in its repeat domain (RD) in HEK293 cells co-transfected with RD-CFP and RD-YFP [36, 37].

**Fig. 5:**
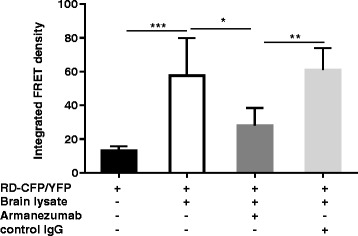
Armanezumab inhibited the seeding activity of pathological tau. **a** Co-incubation with Armanezumab blocked seeding activity of brain lysate of tau (P301S)/Tg mice and significantly decreased the ability to induce the aggregation of RDΔK280-CFP/RD-YFP in transiently transfected HEK293 cells

**Fig. 6:**
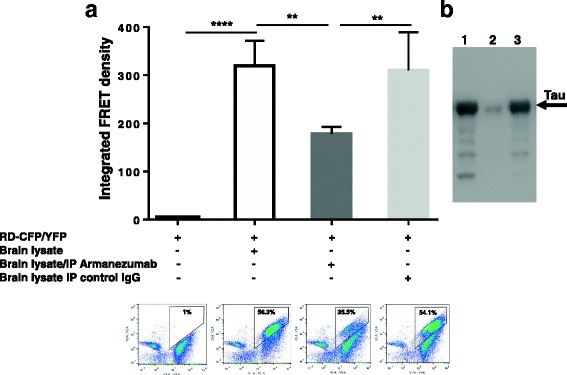
**a** Armanezumab/protein A/G complex bound and removed pathological tau from brain lysate of tau(P301S)/Tg mice significantly decreasing the ability of lysate to induce the aggregation of RD-CFP/RD-YFP in HEK293 cell line constitutively expressing RD-CFP/RD-YFP. FRET positive cells were analyzed by flow cytometry and integrated FRET density was calculated. Representative plots of flow cytometric analyses for each sample are shown. % of FRET positive cells are indicated in plots. **b** Brain lysate of tau(P301S)/Tg mouse immunodepleted with Armanezumab/protein A/G complex or control IgG/protein A/G complex were analyzed by western blot. Bands were visualized using rabbit anti-tau polyclonal antibody. Lane 1-brain lysate; lane 2-brain lysate immunodepleted with Armanezumab; lane 3- brain lysate immunodepleted with control human IgG

More vigorous aggregation could be induced by adding brain lysate from P301S Tg mice containing full-length tau aggregates to the culture of co-transfected cells. Here we tested the ability of Armanezumab to inhibit this brain lysate induction of intracellular aggregation of tau RD containing mutations P301S and ΔK280. As shown in Fig. 5, addition of untreated brain lysate of P301S Tg mice to the transiently transfected cells expressing RD(ΔK280)-CFP/YFP increased the integrated FRET-density, whereas pre-treating brain lysate with Armanezumab inhibited such induction. In another experimental setting, we used monoclonal HEK293T cell lines that constitutively express tau repeat domain (RD) containing P301S mutation tagged to either CFP or YFP. We demonstrated that precipitation of tau from brain lysate with Armanezumab immobilized on Protein G agarose significantly decreased a capacity of brain lysate to induce aggregation of RD-CFP/YFP in these cells and decreased the integrated FRET density (Fig. 6a). Representative WB demonstrating the depletion of tau from brain lysate by immunoprecipitation with Armanezumab/Protein G complex is shown in Fig. 6b. Obviously, in stably transfected cells all of which express RD-CFP/YFP, the number of FRET positive cells, and therefore integrated FRET density, is higher than in transiently transfected cells shown in Fig. 5. These data demonstrated therapeutic potency of Armanezumab, that could not only bind pathological human tau from the brains of Tg mice, but also block seeding activity of aggregated molecules in this *in vitro* assays.

#### Armanezumab inhibits tau cytotoxicity

**Fig. 7:**
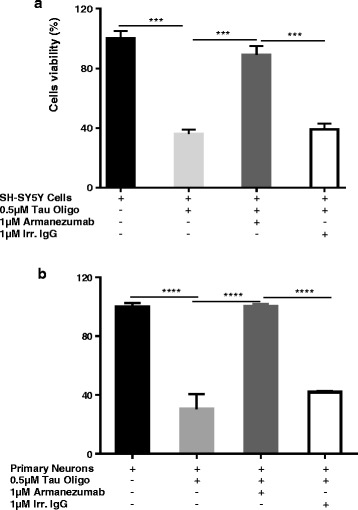
Armanezumab inhibited cytotoxicity of oligomeric recombinant tau protein. **a** SH-SY5Y human neuroblastoma cell line and mouse primary neurons **b** were incubated with tau oligomers in the presence or absence of Armanezumab or control IgG. Control cells were treated with vehicle, and cell viability was assayed in all cultures using the 3-(4,5-dimethylthiazol-2-yl)-2,5-diphenyltetrazolium bromide assay. Data were collected (four replicates) and expressed as percentages of control ± SD

To check the ability of Armanezumab to inhibit neurotoxicity of tau aggregates, we performed cytotoxicity assays to determine whether Armanezumab could protect SH-SY5Y human neuroblastoma cells as well as mouse primary neurons from tau oligomer-mediated neurotoxicity. As seen in Fig. 7, oligomeric tau protein was cytotoxic, reducing neuroblastoma cell viability to 36% and primary neurons viability to 30%, compared to the untreated cells. Pre-incubation of tau oligomers with Armanezumab protected cells from the cytotoxic effects of oligomeric forms of tau, with cell viability reaching 89% and 100% for SH-SY5Y cells and primary neurons, respectively. Of note, the pre-incubation of oligomers with an irrelevant control IgG did not protect neuroblastoma cells or primary neurons from cytotoxicity of this protein (Fig. 7). These data demonstrated potential therapeutic activity of Armanezumab and support testing in *in vivo*.

#### Armanezumab reduced tau in brains of tau/Tg mice

**Fig. 8:**
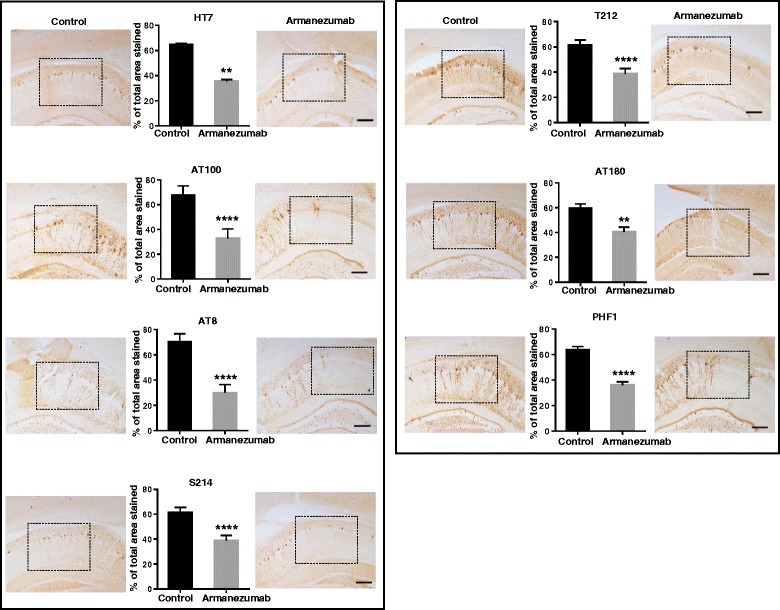
Armanezumab reduced total (HT7) and pThr212, pSer214, PHF1, AT100, AT180, AT8-positive phosphorylated tau in the brains of 6-month-old Thy22-Tau Tg mice after intracranial injection. Quantitative analysis using ImageJ software showed reduced % of stained total area for each ipsilateral region injected with Armanezumab compared to contralateral region injected with control IgG. Bars represent mean ± SD from *n* = 7 mice. Corresponding representative images of injected regions where antibodies diffused after injection, stained with various tau-specific antibodies are shown in the boxed areas for each hemisphere. Original magnifications 10X, scale bar = 100 um

To demonstrate the therapeutic activity *in vivo*, Armanezumab was unilaterally injected into the brains of aged THY-Tau22 Tg mice with tauopathy. Changes in tau pathology were analyzed in brains of mice on the 5th day after antibody administration. Analyses of tau immunoreactivity revealed a substantial decrease of total tau, and tau phosphorylated at positions Thr212, Ser214, Ser396/404 (PHF1), Thr212/Ser214 (AT100), Thr231 (AT180), Ser202/Thr205 (AT8) in ipsilateral hemisphere injected with Armanezumab versus the contralateral hemisphere injected with irrelevant human IgG1 (Fig. 8). Of note, this comparison is based on quantitative image analysis of regions of hippocampus into which antibodies diffused after the administration. These areas were determined by additional staining for human IgG in the adjacent brain sections of mice (data not shown). Additional staining with anti-NeuN antibody confirmed neuronal integrity in the areas around the injection sites (data not shown). These data suggest that Armanezumab is able to significantly reduce pathological tau in the brains of tau/Tg mice with established tauopathy after intracranial administration.

### Pharmacokinetics of Armanezumab in mice and development of industrial cell line

**Fig. 9:**
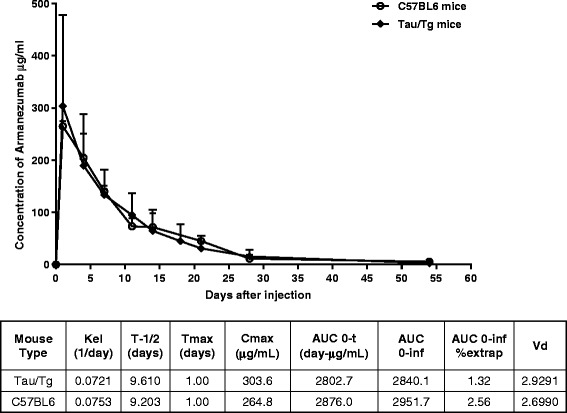
Mean serum concentrations and pharmacokinetics parameters of Armanezumab following a single IV dose to PS19 tau/Tg and C57BL6 mice. Error bars represent average ± SD (*n* = 3 for C57BL6 and *n* = 9 for PS19 tau/Tg groups). Pharmacokinetic analyses were performed as described in Materials and Methods. Kel, elimination constant; T-1/2, elimination half-life; Tmax, the time to reach max concentration; Cmax, the maximum concentration; AUC 0-t, area under the concentration-time curve; AUC 0-inf, the area to infinity; AUC 0-inf %extrap, % of extrapolated AUC to infinity; Vd, the apparent volume of distribution

PK analyses of Armanezumab in wildtype mice, in the absence of target-mediated clearance and in Tg mouse model expressing human tau, was conducted after the administration of a single dose of Armanezumab via IV bolus injection to C57BL6 and PS19 tau/Tg mice. The concentration of Armanezumab was monitored from day 1 through day 54 following the injection. No differences were observed in the serum concentration x time profiles of Armanezumab in WT and PS19 mice (Fig. 9). In both strains the peak plasma concentration (C_max_) of Armanezumab was observed at day 1, and the average elimination half-life is 9 days (Table in Fig. 9). The rate at which Armanezumab is removed from the system [Elimination rate (K_el_) constant and the estimated clearance (CL)] also did not differ between strains indicating that the elimination was not target-mediated. The apparent volume of distribution is 2.93 and 2.70 in Tg and wild-type mice, respectively, indicating that Armanezumab was mostly distributed in blood in both strains of mice.

Based on data generated in the studies described above, a stable CHO‐DG44 cell line expressing quantities of Armanezumab amenable to scale-up production (1.5 g/L), was developed using MTX amplification.

## Discussion

Although Aβ may be the primary trigger in AD pathogenesis, it is clear that pathological tau also plays an important role in AD [38]. By the time the clinical signs of AD appear first, there is already substantial tau pathology in the brain [39, 40], which may become self-propagating [41–44]. Observations showed that tau oligomers are directly toxic to neurons, and tau pathology better correlates with clinical cognitive decline in AD [14, 45, 46]. Therefore, several groups have proposed anti-tau immunotherapy (passive and active vaccinations) as an effective therapeutic approach [4, 47–49].

Importantly, therapeutics aimed at eliminating pathological tau may also be beneficial for treatment of a group of neurodegenerative disorders other than AD, categorized as tauopathies. These diseases include ALS, FTD with parkinsonism linked to chromosome 17, Pick’s Disease, PSP, Creutzfeldt-Jakob Disease, Dementia Pugilistica, Down’s Syndrome and others.

Tau-targeting immunotherapy was questioned initially due to the intracellular nature of tau protein. However, data demonstrating that during disease progression in humans tau-related pathology spreads from the affected areas of the brain to the healthy regions [50], and the discovery of trans-cellular propagation of tau aggregates [51–55] suggested a possible mechanism of action for antibody-mediated reduction of tau pathology observed in mouse models, and supported the feasibility of using anti-tau immunotherapy in a clinical setting. Although the exact mechanism of antibody-mediated reduction of pathological tau is currently unknown, several studies suggest different mechanisms of action, possibly depending on the particular antibody, as well as type and size of tau aggregates [56–61]. Recently it was shown that tau aggregates-antibody complexes taken up by cultured neuronal cells bind to cytosolic protein TRIM21 that triggered clearance of tau particles [58]. A mechanism of action where neuronal FcγRs mediate antibody uptake by neurons, followed by engagement of intracellular tau tangles by the internalized antibody, has been proposed [56, 57]. Another suggested mechanism is that antibodies bind extracellular Tau and prevent its uptake by adjacent or post-synaptic neurons [59, 60]. It is also shown that anti-tau antibodies can promote microglial uptake and clearance of tau [59, 61], although, recently another group reported that effector function is not required for antibody efficacy [60].

An early immunotherapeutic study showed that immunization of wild type mice with recombinant full-length tau is not entirely safe, and tau-specific polyclonal antibodies induced NFT-like pathology, glial activation, axonal damage, and even infiltration of mononuclear cells into the brains [62]. However, several subsequent studies utilizing both passive vaccinations [43, 57, 63–72] and short peptide-based active vaccinations [73–79] targeting various phosphorylated, non-modified linear or conformational epitopes of tau showed that these antibodies are safe and efficacious for reducing tau pathology and improving tau-associated functional deficits in tau/Tg mouse models. Currently, two active vaccines (AADvac1 [80, 81] and ACI-35 [79]) are being tested in clinical trials with AD patients, and two humanized versions of anti-tau antibodies have been reported to have advanced to clinical testing in subjects with progressive supranuclear palsy (NCT02494024, NCT02460094, NCT02658916).

In this study, we report a discovery and characterization of a humanized antibody targeting N-terminal region of tau, which, after successful preclinical safety studies will be ready to advance into clinical testing. As our starting point, a mouse mAb was selected based on the ability of binding to recombinant tau with high affinity, as well as binding to oligomeric/fibrillar tau in brain homogenates and NT/NFT in the brain tissue from AD cases. Importantly, our data reveals this mAb’s high specificity to pathological tau species characterized by an accessible N-terminal region, and it’s inability to effectively bind normally folded tau in non-diseased brain. Translation of monoclonal antibodies to a clinical setting requires humanization to make them non-immunogenic or at least less immunogenic in humans. For humanization of mouse mAb 1C9, we adopted an approach involving CDR-grafting into selected human frameworks from human Immunoglobulin Germline V gene database based on similarity to the parent mouse mAb, combined with phage display to identify the optimal humanized variant. Constructs encoding humanized VH and VL were fused with constant regions from IgG1 human immunoglobulin to generate full human antibody. The optimal humanized antibody (Armanezumab) was characterized in different *in vitro*/*ex vivo* and *in vivo* assays to test for activity relative to the parent mouse mAb. We demonstrated that Armanezumab (i) possesses a tau-binding affinity, which is only slightly lower than parental mouse mAb 1C9; (ii) recognizes the same tau_4–8_ epitope as 1C9; (iii) efficiently binds to pathological tau in brain sections from AD, FTD, Pick’s Disease cases, without binding to brain sections from control non-demented subjects; (iv) significantly inhibits the seeding ability of misfolded tau in brain extracts from P301S Tg as evidenced by decreasing integrated FRET density in a cellular assay of tau aggregation; (v) Inhibits neurotoxic effects of aggregated tau; vi) acutely reduces tau pathology following intracranial administration in Tau Tg mice.

In addition, pharmacokinetics analysis of Armanezumab revealed no differences in clearance rates from the blood circulation of wildtype and tau/Tg PS19 mice, with about 9 days of half-life in both strains. This similarity in pharmacokinetics of mouse and humanized Mab could be explained by negligible concentrations of tau protein in blood of tau transgenic mice that express human tau selectively in neurons, leading to the same non-target-mediated profile of elimination seen in wild-type mice. Despite in general higher binding affinity of human Fc-fragment to mouse neonatal FcRn [82], half-life of Armanezumab is close to theoretical mouse IgG half-life. In fact, it was shown that higher affinity for FcRn does not correlate with extended *in vivo* half-lives of antibodies [83].

Recently, various groups developed antibodies specific to different epitopes located at N-terminal region of Tau. It was demonstrated that both intracerebroventricular infusion and peripheral administration of antibodies specific to Tau_25–30_ are therapeutic in P301S mouse model of tauopathy [43, 72], although very high concentration (50 mg/kg) of systemically administered (i.p.) antibodies are needed to achieve significant decrease in insoluble tau, prevent brain atrophy, and see improvement of motor/sensorimotor function [72]. On the other hand, intravenous injection of only 15 μg (~750 μg/kg) of antibody specific to Tau_6–18_ markedly reduced tau pathology and rescued cognitive impairment of vaccinated 3xTg-AD mice [69, 84]. Such large differences in effective dose of above mentioned antibodies could be attributable to differences in administration route, targeted epitope or the aggressiveness of mouse model of tauopathy. This assumption is supported by another study showing that single i.v. administration of 60 μg antibodies specific to oligomeric tau for 60 d was sufficient to reverse both locomotor and memory deficits in a not very aggressive Htau mouse model of tauopathy [85].

Overall, these data suggest that ideally various routes of delivery, variable doses and different mouse models should be tested to properly assess the antibody’s ability to reduce the tau pathology. However, even after such careful testing it is difficult to predict the efficacy of antibodies in humans. The majority of mouse models of tauopathies constitutively overexpress mutated human tau and rapidly develop severe pathology, although not completely resembling human disease. We believe that the modest efficacy of many anti-tau antibodies currently tested in preclinical studies might be associated with very low amount of antibody reaching CNS for clearing such severe pathology. Therefore, various groups suggest testing the efficacy of anti-tau biologics and drugs in *in vitro* or *ex vivo* experimental systems such as (i) reduction of aggregation of tau; (ii) inhibition of toxicity of oligomeric tau and inhibition of propagation of tau [86]. As mentioned above, the efficacy of Armanezumab was shown in all these assays, therefore, to save time and reduce the cost of non-clinical studies we decided to evaluate the ability of Armanezumab to reduce pathological tau after intracranial injection of mice. These *in vivo* data demonstrated that 2 μg/mouse of Armanezumab in 5 days could reduce not only total tau, but also various pathological tau molecules phosphorylated at positions S212, S214, Ser396/404 (PHF1), Thr212/Ser214 (AT100), Thr231 (AT180), Ser202/Thr205 (AT8). Fine epitope mapping revealed the epitope PRQEF at positions tau 4–8 with the most essential amino acids at positions 6&7, indicating that Armanezumab is different from antibodies specific to N-terminal tau region reported previously by other groups [43, 69]. Based on the fact that only a tiny amount of circulating antibody passes the BBB and enters the brain [87–89] we think that more feasible route of administration for antibodies might be intranasal route as a non-invasive and efficient approach of delivering therapeutics to the brain that circumvents systemic extraction/alteration [90, 91].

## Conclusions

Overall, we have generated and successfully humanized a murine mAb specific to N-terminus of tau protein. The humanized antibody, designated Armanezumab, recognized NTs and NFTs in brain tissue from AD, FTD and Pick’s Disease cases, inhibited seeding activity and neurotoxicity of aggregated tau and reduced pathological tau in brains of THY-Tau22 transgenic mice. A CHO cell line expressing Armanezumab amenable to scale up production (1–2 g/L) has been developed to support IND enabling studies with Armanezumab, as well as advancement to phase 1 clinical testing.

## Methods

### Mice, peptide epitope vaccine, and immunizations

Female, 6–8 weeks old B6SJL (H-2^bxs^ haplotype) and PS19 Tau/Tg mice were obtained from the Jackson Laboratory. THY-Tau22 Tau/Tg mice (generous gift of Dr. Luc Buee, Inserm) were bred at UCI animal facility. All animals were housed in a temperature and light-cycle controlled facility, and their care was under the guidelines of the National Institutes of Health and an approved IACUC protocol at University of California, Irvine. B6SJL mice were immunized with MultiTEP platform-based vaccine, AV-1980R, as previously described [92]. Briefly, mice were injected subcutaneously (SC) with 50 μg/mouse dose of AV-1980R formulated in Quil-A adjuvant (Sigma-Aldrich, MA). All mice were boosted at 2-week intervals. Sera were collected on day 12 after each immunization. Mice were terminated 4 days after the 4^th^ immunization and splenocytes were used for fusion and selection of hybridomas producing anti-Tau antibodies.

Peptides were synthesized by standard chemical peptide synthesis and purity was analyzed by HPLC (GenScript, NJ).

### Generation of hybridoma

Mouse spleen was aseptically removed, cells were washed out from the spleen by perfusion with 10 ml of sterile serum-free DMEM/F12. Splenocyte suspension was passed through sterile Falcon 0.70 μm cell filter followed by two wash steps. Splenocytes were fused with SP2/0 myeloma (Sp2/0-Ag14 ATCC® CRL-1581) cells per standard protocols and selected in HAT media. Hybridoma supernatants were screened by ELISA for reactivity with tau_2–18_ peptide. Positive hybridomas were subcloned by limiting dilution. Fusion procedure and subcloning have been performed in Recombinant Protein Production Core (rPPC), CLP at Northwestern University. Twenty-eight clones were selected and screened for binding to brain sections from the AD case. Clone 1C9 has been selected and used for further characterization and humanization.

### Monoclonal antibody sequencing

Total RNA was isolated from hybridoma cells following the technical manual of TRIzol® Plus RNA Purification System and analyzed by agarose gel electrophoresis. RNA was reverse transcribed into cDNA using isotype-specific anti-sense primers or universal primers following the technical manual of SuperScriptTM III First-Strand Synthesis System. The antibody fragments of VH, VL, CH and CL were amplified according to the standard operating procedure for RACE at GenScript (Genscript, Piscataway, NJ). Amplified antibody fragments were separately cloned into a standard cloning vector using standard molecular cloning procedures. Clones with inserts of correct sizes were identified by PCR screening. No less than five single colonies with inserts of correct sizes were sequenced for each antibody fragment.

### Humanization of mouse antibody

1C9 antibody was humanized by Genscript using proprietary technology. Variable domain sequences were blasted against human germline and several FR1, FR2, and FR3 were selected independently from human FRs, which share the highest identity with the mouse antibody. Selected FRs were assembled with 1C9 CDRs using overlapping PCR and phage display library was constructed for expression of Fab fragments. High tau protein-binding phages were selected after three rounds of panning using Genscript’s proprietary FASEBA screening methodology. Selected Fab genes were amplified from phage DNA. Genes encoding Fab were fused with genes encoding the appropriate constant regions of human IgG1 in order to generate whole IgG. Resulting light chain and heavy chain constructs were cloned into the mammalian expression vectors pcDNA3 and pGN-M, respectively.

### Purification of Armanezumab

Stable DG44 cell line expressing Armanezumab mAb was generated by clone selection with increased concentrations of methotrexate (MTX). DG44 cells of selected clone were re-suspended at a density of 5x10^5^ cells/ml and 600 ml of cell suspension was seeded into 1 L shaker flasks. 10% (v/v) Feed B solution was added to the culture on day 2, day 4, day 6, day 8. Glutamine was added to maintain the concentration range from 1 mM to 4 mM, and glucose was added to the culture to maintain the concentration range from 4 g/L to 10 g/L. Cell counting was conducted daily to determine the cell density and viability. The collected cell culture supernatants were used for purification when the cell viability was below 50%. Cell culture broths were centrifuged and followed by filtration. Filtered supernatant was loaded onto Protein A CIP column 65 ml (GenScript, Cat.No.L00433) at 8.0 ml/min. After washing with PBS, elution with 50 mM Citric acid, pH 3.0 and neutralization with 1 M Tris–HCl, pH 9.0, the eluted fractions were desalted by HiPrep 26/10 Desalting column with PBS. The purified antibody was analyzed by SDS-PAGE, Western-blot and HPLC by using standard protocols for molecular weight, yield and purity measurements.

### Epitope mapping of tau-specific antibodies

Epitope mapping of anti-tau antibodies was performed by “alanine scanning” using competitive ELISA. Briefly, 17 peptides spanning tau_2–18_ sequence, but possessing one alanine substitution in each position were synthesized. 96-well plates (Immulux HB; Dynex Technologies, Inc., VA) were coated with 1 μg/well (in 100 μl; Carbonate-Bicarbonate buffer, pH 9.6, o/n at 4 °C) tau_2–18_ peptide (GenScript, NJ). Next day coated plates were blocked with blocking buffer (3% dry, non-fat milk in TBST, 300 μl/well). Serial dilutions of reference wild type (tau_2–18_), or mutated test peptides (corresponding to 0.02 μM, 0.1 μM, 0.5 μM, 2.5 μM and 12.5 μM final concentrations) were incubated with anti-tau antibodies (0.04 mg/ml final concentration) for 1.5 h at 37 °C. After incubation 100 μl of antibody/peptide mixture were added into the wells. HRP-conjugated goat anti-mouse IgG (1:2500; Jackson ImmunoResearch Laboratories, PA) in case of 1C9 and HRP-conjugated goat anti-human IgG (1:2500; Jackson ImmunoResearch Laboratories, PA) in case of Armanezumab were used as secondary antibodies. The reaction was developed by adding *3,3’,5,5’tetramethylbenzidine* (TMB) (Pierce, IL) substrate solution and stopped with 2 M H_2_SO_4_. The optical density (OD) was read at 450 nm (Biotek, Synergy HT, VT). The percent of binding of 1C9 and Armanezumab antibodies blocked with wild type or mutated peptides to tau_2–18_ was calculated relative to the binding of 1C9 and Armanezumab antibodies without competing peptides to tau_2–18_ as 100%. The half maximal inhibitory concentration (IC_50_) for each peptide was calculated by Excel.

### Recombinant tau protein preparation

Gene encoding 0N4R tau protein was amplified from human whole brain Marathon®-Ready cDNA library (Clontech) using primers 5’-*catatg*gctgagccccgccaggagttcgaagtgatg (forward) and 5’-*ctcgag*tcacaaaccctgcttggccagggaggcagac (reverse) and cloned into the pET24a + E.coli expression vector in frame with 6xHis-tag at the C-terminus using restriction sites NdeI and XhoI. The sequence of the cloned tau protein was verified by DNA sequence analysis. Gene encoding tau, containing deletion of 2–18 region was amplified from plasmid carrying full-length tau gene and cloned into the pET24a + vector. Both recombinant proteins were purified from *E. coli* BL21 (DE3) cells transformed with pET24a+/tau or pET24a+/tauΔ2–18 plasmids. Cells were grown up to the optical density (OD) 0.7–0.8 at 600 nm in the Luria Bertani medium containing 100 μg/ml of kanamycin. Gene expression was induced by adding isopropyl-β-D-1-thiogalactopyranoside (IPTG) at a final concentration of 1 mM and incubating for 4 h at 28 °C. Cells harvested by centrifugation were re-suspended in the B-PER reagent (Pierce) disrupting the integrity of cells. NaCl was added to a final concentration of 500 mM to the lysate and solution was boiled for 20 min. Most of the proteins precipitated following heat denaturation, while tau remained in the solution. Denatured proteins were removed by centrifugation and 6xHis-tagged tau was purified from the final supernatant using Ni-NTA columns (Qiagen, CA). Positive fractions were combined and concentrated by centricon filters (Millipore, MA). Concentrated protein fractions were analyzed by 10% Bis-Tris gel electrophoresis (NuPAGE Novex Gel, Invitrogen, CA) (Fig. 1).

### Preparation of oligomeric and fibrillar tau

Fibrillar and oligomeric forms of tau (cross-linked and non-cross-linked) have been kindly provided by Dr. Kayed (UTMB Neurology). Oligomeric Tau used in neurotoxicity assay were prepared according to protocol described in [93], using seed oligomers obtained from Dr. Kayed. Briefly, 7 μl of tau oligomers 0.6 mg/ml were added to 1 ml of recombinant tau protein solution of the same concentration and incubated for 1, 2, 4.5 and 24 h on an orbital shaker.

### Preparation of human and mouse brain homogenates

Frozen blocks of post mortem human brains from AD (Braak stage VI) and control (Braak stage 0) cases were received from the Brain Bank and Tissue Repository, MIND, University of California, Irvine. 0.2 g of each brain tissue were homogenized in 0.4 ml TBS buffer with Halt™ Protease and Phosphatase Inhibitor Cocktail (100X, Thermo Scientific, CA), then centrifuged at 6400xg for 15 min at +4 °C. Supernatants were collected and stored at−80 °C for further analysis as soluble fractions.

Mouse brain homogenates were prepared exactly as described in [37], More specifically, brain tissue was suspended in 10% (wt/vol) ice-cold TBS containing cOmplete protease inhibitors (Roche). Tissue was homogenized at 4 °C using a probe sonicator (Omni Sonic Ruptor 250) at 30% power, receiving 25 pulses. Lysates were centrifuged at 21,000 × g for 15 min to remove cellular debris and large, insoluble material. Supernatants were aliquoted and stored at−80 °C until further use.

### Western blot

Western Blot analyses of recombinant full-length 4R/0 N tau, tauΔ2–18 and soluble fractions from brain homogenates were performed to confirm the specificity and binding ability of 1C9 and Armanezumab to pathological human tau. Commercial HT7 and TNT-1 antibodies have been used as positive controls. Briefly, brain homogenates containing equal amounts of total protein in SDS sample buffer (non-reducing conditions without heat incubation) were subjected to electrophoresis in 10% Bis-Tris polyacrylamide gel in MES buffer (Invitrogen, CA), then electro-transferred onto nitrocellulose membrane (GE Healthcare, NJ). The membranes were blocked overnight with 5% fat-free dry milk in TBS with 0.05% Tween following by detection of tau using 1C9, Armanezumab, HT7 (Life Technology, CA) or TNT-1 (Millipore, MA) monoclonal antibody and appropriate HRP-conjugated secondary antibody. All primary antibodies were used at concentration 1 μg/ml. Proteins were visualized with enhanced chemiluminescence detection using Luminol reagent (Santa Cruz Biotechnology, CA).

### Dot blot

#### Binding to recombinant tau

1 μl of each form of tau protein (monomeric, oligomeric and fibrillar) was applied to nitrocellulose membrane (GE Healthcare, NJ). The membrane was air-dried, blocked overnight with 5% fat-free dry milk in TBS with 0.05% Tween following by detection of tau using 1C9 monoclonal antibody (1 μg/ml) and HRP-conjugated anti-mouse secondary antibody (0.2 μg/ml).

#### Binding to tau in human brain tissues

Soluble fractions of brain extracts from AD cases and controls were applied to membrane (2 μg of total protein in 1 μl volume) and proteins were detected using mouse 1C9, TNT-1 (Millipore, MA), HT7 (Life Technology, CA) monoclonal and rabbit polyclonal anti-Tau antibodies (BioLegend, CA). All primary antibodies were used at concentration of 1 μg/ml. Bovine anti-mouse and mouse anti-rabbit HRP-conjugated secondary antibodies were used at concentration of 0.2 μg/ml (Santa Cruz Biotechnology, CA). Proteins were visualized with enhanced chemiluminescence detection using Luminol reagent (Santa Cruz Biotechnology, CA).

### Binding of Armanezumab to pathological tau in human brain tissues

1C9 or Armanezumab (1.0 μg/ml) were tested for the ability to bind to tau tangles in the human brain using 50 μm brain sections of formalin-fixed cortical tissues from severe AD (inferior parietal gyrus, *n* = 6), FTD (midfrontal cortex, *n* = 3) and Pick’s Disease (midfrontal cortex, *n* = 3) cases and normal control brains (inferior parietal gyrus, *n* = 2), all received from Brain Bank and Tissue Repository, MIND, UC Irvine, using immunohistochemistry as described previously [92]. A digital camera (Olympus) was used to capture the representative images at 40x (for IC9) and 60x (for Armanezumab, PHF1, AT8, AT100, TNT-1, and HT7) original magnifications.

### Surface plasmon resonance (SPR) analyses

SPR binding studies were performed on the BIAcore T200. Antibodies were immobilized on the surface of the biosensor chip CM5 series S (GE Healthcare) through Fc capture. Serial dilutions of tau recombinant protein in the running buffer containing 10 mM HEPES, 150 mM NaCl, 3 mM EDTA, 0.005% Tween 20, pH 7.4, were injected at 5 mL/min over immobilized antibody, and the kinetics of binding/dissociation were measured as a change in the SPR signal (in resonance units). Each injection was followed by a regeneration step consisting of a 12-s pulse of 50 mM HCl. Fitting of experimental data was done with Biacore T200 evaluation software, version 1.0 using a 1:1 interaction model to determine apparent binding constants.

### Blocking of brain lysate seeding activity

Measuring of RD-CFP/YFP co-aggregation by FRET was described in detail previously [36, 37]. Briefly, HEK293 cells were plated at 250,000 cells/well in a 12-well plate and co-transfected with plasmids encoding P301S tau repeat domain (RD) containing ΔK280 mutation and fused to cyan (RD(ΔK280)-CFP) or yellow (RD(ΔK280)-YFP) fluorescent protein at proportions 1:3 as described earlier. 15 h later, cells were harvested with 0.05% trypsin for 3 min at 37 °C, and then re-plated in a 96-well plate in quadruplicate. After 15 h brain lysates of P301S Tg mice untreated (prepared in 1X TBS with protease, phosphatase inhibitors) or pre-incubated for 16 h at 4 °C under constant rotation with Armanezumab (10 μg/ml) were added. Cells were then cultured for additional 24 h before FRET analysis by flow cytometry. For determination of baseline level of RD(ΔK280)-CFP/YFP endogenous aggregation, cells were cultured without addition of brain lysates or antibody. FRET flow cytometry was performed using MACSQuant VYB (Miltenyi). Integrated FRET density was calculated as percent of FRET-positive cells x median fluorescence intensity (MFI).

### Brain lysate seeding activity after immunodepletion

Armanezumab and control antibodies (50 μg) were added to agarose beads with protein A and G, then covalently coupled using Crosslink Immunoprecipitation Kit (Pierce) according to the provided protocol. Then brain lysates were immunodepleted four times with column elution between each immunodepletion. The final immunodepleted lysate was normalized to 0.97 μg/ml protein (measured by BCA), co-incubated with lipofectamine® reagent and added to HEK293T cell line (kindly provided by Dr. Diamond) constitutively expressing P301S tau repeat domain (RD) fused with either cyan protein (CFP) or yellow protein (YPF). Overall 10.3 μl of lysate, 1.25 μl of lipofectamine and 13.45 μl of optimem were used for each well. The incubations of lipofectamine were done according to the recommended protocol for DNA transfection. After 24 h incubation cells were harvested by trypsinization and tested for FRET by flow cytometry. Integrated FRET density was calculated.

### Neurotoxicity assay

Neurotoxicity of oligomeric tau was assessed in SHSY-5Y cell culture and in primary neurons using MTT (3-[4,5-dimethylthiazol-2-yl]-2.5-diphenyltetrazolium bromide) assay performed as described previously [31, 32] in the presence of tau oligomers plus or minus test antibody (Armanezumab). Incubation time of cells with tau oligomers and tau oligomers/antibodies was 16 h, and the final concentrations of protein and antibodies were 0.5 μM and 1 μM, respectively.

### Intracranial injection of antibodies and quantitative image analysis

Armanezumab and control IgG were delivered to the hippocampus and cortex using a stereotaxic apparatus. Briefly, tau/Tg mice were anesthetized with isofluorane, placed in the stereotaxic frame and injected with Armanezumab (2 μg/μl) into the hippocampus (1 μl/injection) and into the cortex (1 μl/injection) by a 5 μl Hamilton microsyringe (30-gauge) at an injection rate of 0.5 μl/min, using the following coordinates relative to Bregma: AP: −2.06, ML: ±1.75; DV: −1.95 pocket 1 μl, and −1.00 1 μl. Control IgG was injected into the contralateral hemisphere using the same technique. The right side of each brain received Armanezumab while the left side was injected with the control IgG. Mice were sacrificed five days after the injection via Nembutal overdose and transcardially perfused with ice-cold 0.01 M phosphate-buffered saline (PBS). Brains were rapidly removed and fixed in 4% paraformaldehyde in PBS (pH 7.4) for 24 h at 4 °C and then sunk in 20% sucrose (in PBS). Prior to sectioning, a notch was made in the bottom left cortex so that left and right sides could be readily distinguished after sectioning. Brains were then cut in 40 μm thick coronal sections on a Microtome slicing system (Leica) and stored in PBS with 0.05% NaN3 at 4 °C until used. Sections were mounted on the slides and dried. Slices were pretreated with sodium citrate buffer, pH 6.0 at 90 °C for 30 min. All sections were hydrogen peroxide quenched, blocked and incubated with primary antibody overnight at 4 °C. Total tau was detected with HT7 recognizing epitopes 159–163, (0.4 μg/ml), phosphorylated tau was detected with T212 (pT212, 1 μg/ml), S214 (pS214, 0.4 μg/ml), AT100 (pT212/pS214, 0.4 μg/ml), PHF1 1 μg/ml (pS396/pS404, 2 μg/ml), AT8 (pS202/T205, 0.4 μg/ml) (all from ThermoFisher Scientific, Waltham, MA except for PHF1 from Peter Davis), and AT180 (pT231, 0.4 μg/ml (Abcam, Cambridge, United Kingdom). Anti-human IgG antibody was used to determine the antibody diffusion area in the brain parenchyma and anti-NeuN antibody was used to define the neuron density in injected sites. Incubations with appropriate biotinylated secondary antibody and ABC for 1 h were performed followed by color development using DAB (3,3’-diaminobenzidine) substrate kit (both from Vector Labs, CA). Images were captured by an Olympus microscope. Immunostaining profiles were observed by the means of a Sony high resolution CCD video camera (XC-77) and NIH Image version 1.59b5 software. For every animal, the images of the CA1 areas of three sections per each antibody were captured with the 10X objective. For the quantitative analysis, the images were cropped to the size of 1000 × 800 pixels at 600 dpi resolution, imported using ImageJ software (Scion) and converted to black and white using automatic binarization script. Thresholds were calculated for each picture separately using the same algorithm, and the number of pixels expressing staining density was determined in CA1 (injection) area for both ipsilateral and contralateral hemispheres. Density is expressed in the percentage units calculated using formula I/(I + C)x100 and C/(I + C)x100 where I-ipsilateral, C-contralateral. Average and standard deviation for each hemisphere between all mice were calculated and compared.

### Pharmacokinetic data analysis

C57BL6 and PS19 tau/Tg mice received a single 600 μg/mouse intravenous (IV) dose of Armanezumab. Blood samples were collected by retro-orbital bleeding for PK at the following time points: 1, 4, 7, 11, 14, 18, 21, 28 and 54 days. Three mice per group underwent bleeding for each time point so that blood was drawn from an individual mouse only for every fourth time point. Concentrations of Armanezumab in the sera were determined by ELISA as described in [94] except that plates were coated with 2.5 μM recombinant tau protein and purified 1C9 mAb and Armanezumab were used for calibration curve. PK parameters were calculated by non-compartmental analysis of the mean concentration values for each mouse using WinNonlin, version 5.2 (Pharsight). The following standard pharmacokinetic parameters were determined: (i) the maximum concentration (C_max_); (ii) the time of C_max_ occurrence (t_max_); (iii) area under the sera concentration-time curve from time zero to the time of the last measured serum level (AUC); (iv) t_1/2_, calculated by linear regression of the logarithm of plasma concentration-time curve; (v) elimination constant kel = 0.693/t_1/2_; (vi) apparent volume of distribution (V_d_) obtained by calculating V_d_ = dose/AUC x k_el_.

### Statistical analysis

All statistical parameters [mean, standard deviation (SD), significant difference, etc.] used in experiments were calculated using Prism 6 software (GraphPad Software, Inc.). Statistically significant differences were examined using unpaired *t*-test or Ordinary one-way ANOVA Tukey’s multiple comparisons test (*P* value less than 0.05 was considered as statistically different).

## Abbreviations

AD: Alzheimer’s disease
Aβ: amyloid-β
B-PER: bacterial protein extraction reagent
CDR: complementarity determining region
CFP: cyan fluorescence protein
CHO: Chinese hamster ovary
ELISA: enzyme-linked immunosorbent assay
FASEBA: fast screening for expression, biophysical and affinity
FAT: fast axonal transport
FR: framework region
FRET: fluorescence resonance energy transfer
Fv: variable fragment
HEK293: human embryonic kidnay 293 cells
HEPES: 4-(2-hydroxyethyl)-1-piperazineethanesulfonic acid
HPLC: high pressure liquid chromatography
HRP: horseradish peroxidase
IHC: immunohistochemistry
mAb: monoclonal antibody
MES: 2-(N-morpholino)ethanesulfonic acid
MTT: 3-(4,5-dimethylthiazol-2-yl)-2,5-diphenyltetrazolium bromide
MTX: *methotrexate*
NFT: neurofibrillary tangles
Ni-NTA: nickel-nitrilotriacetic acid
NT: neuropil threads
OD: optical density
PAD: phosphatase activation domain
PBS: phosphate buffered saline
RD: repeat domain
SDS: sodium dodecyl lsulfate
SPR: surface plasmon resonance
TMB: 3,3’,5,5’-tetramethylbenzidine
YFP: yellow fluorescence protein.

## References

[1] Giacobini E, Gold G (2013). Alzheimer disease therapy--moving from amyloid-beta to tau. Nat Rev Neurol.

[2] Schneider LS, Mangialasche F, Andreasen N, Feldman H, Giacobini E, Jones R, Mantua V, Mecocci P, Pani L, Winblad B, Kivipelto M (2014). Clinical trials and late-stage drug development for Alzheimer’s disease: an appraisal from 1984 to 2014. J Intern Med.

[3] Winblad B, Graf A, Riviere ME, Andreasen N, Ryan JM (2014). Active immunotherapy options for Alzheimer’s disease. Alzheimers Res Ther.

[4] Wisniewski T, Goni F (2014). Immunotherapy for Alzheimer’s disease. Biochem Pharmacol.

[5] Lobello K, Ryan JM, Liu E, Rippon G, Black R (2012). Targeting Beta amyloid: a clinical review of immunotherapeutic approaches in Alzheimer’s disease. Int J Alzheimers Dis.

[6] Lannfelt L, Relkin NR, Siemers ER (2014). Amyloid-β-directed immunotherapy for Alzheimer’s disease. J Intern Med.

[7] Agadjanyan MG, Petrovsky N, Ghochikyan A (2015). A fresh perspective from immunologists and vaccine researchers: Active vaccination strategies to prevent and reverse Alzheimer’s disease. Alzheimers Dement.

[8] Berg L, McKeel DW, Miller JP, Storandt M, Rubin EH, Morris JC, Baty J, Coats M, Norton J, Goate AM (1998). Clinicopathologic studies in cognitively healthy aging and Alzheimer’s disease: relation of histologic markers to dementia severity, age, sex, and apolipoprotein E genotype. Arch Neurol.

[9] Dickson DW, Crystal HA, Bevona C, Honer W, Vincent I, Davies P (1995). Correlations of synaptic and pathological markers with cognition of the elderly. Neurobiol Aging.

[10] Bierer LM, Hof PR, Purohit DP, Carlin L, Schmeidler J, Davis KL, Perl DP (1995). Neocortical neurofibrillary tangles correlate with dementia severity in Alzheimer’s disease. Arch Neurol.

[11] Iqbal K, Alonso AC, Gong CX, Khatoon S, Pei JJ, Wang JZ, Grundke-Iqbal I (1998). Mechanisms of neurofibrillary degeneration and the formation of neurofibrillary tangles. J Neural Transm Suppl.

[12] Augustinack JC, Schneider A, Mandelkow EM, Hyman BT (2002). Specific tau phosphorylation sites correlate with severity of neuronal cytopathology in Alzheimer’s disease. Acta Neuropathol.

[13] Nelson PT, Jicha GA, Schmitt FA, Liu H, Davis DG, Mendiondo MS, Abner EL, Markesbery WR (2007). Clinicopathologic correlations in a large Alzheimer disease center autopsy cohort: neuritic plaques and neurofibrillary tangles “do count” when staging disease severity. J Neuropathol Exp Neurol.

[14] Nelson PT, Alafuzoff I, Bigio EH, Bouras C, Braak H, Cairns NJ, Castellani RJ, Crain BJ, Davies P, Del Tredici K (2012). Correlation of Alzheimer disease neuropathologic changes with cognitive status: a review of the literature. J Neuropathol Exp Neurol.

[15] Jeganathan S, von Bergen M, Brutlach H, Steinhoff HJ, Mandelkow E (2006). Global hairpin folding of tau in solution. Biochemistry.

[16] Ward SM, Himmelstein DS, Lancia JK, Binder LI (2012). Tau oligomers and tau toxicity in neurodegenerative disease. Biochem Soc Trans.

[17] Morfini GA, Burns M, Binder LI, Kanaan NM, LaPointe N, Bosco DA, Brown RH, Brown H, Tiwari A, Hayward L (2009). Axonal transport defects in neurodegenerative diseases. J Neurosci.

[18] Horowitz PM, Patterson KR, Guillozet-Bongaarts AL, Reynolds MR, Carroll CA, Weintraub ST, Bennett DA, Cryns VL, Berry RW, Binder LI (2004). Early N-terminal changes and caspase-6 cleavage of tau in Alzheimer’s disease. J Neurosci.

[19] Gamblin TC, Berry RW, Binder LI (2003). Tau polymerization: role of the amino terminus. Biochemistry.

[20] Kanaan NM, Morfini G, Pigino G, LaPointe NE, Andreadis A, Song Y, Leitman E, Binder LI, Brady ST (2012). Phosphorylation in the amino terminus of tau prevents inhibition of anterograde axonal transport. Neurobiol Aging.

[21] Kanaan NM, Morfini GA, LaPointe NE, Pigino GF, Patterson KR, Song Y, Andreadis A, Fu Y, Brady ST, Binder LI (2011). Pathogenic forms of tau inhibit kinesin-dependent axonal transport through a mechanism involving activation of axonal phosphotransferases. J Neurosci.

[22] Combs B, Hamel C, Kanaan NM (2016). Pathological conformations involving the amino terminus of tau occur early in Alzheimer’s disease and are differentially detected by monoclonal antibodies. Neurobiol Dis.

[23] Zhao Y, Tseng IC, Heyser CJ, Rockenstein E, Mante M, Adame A, Zheng Q, Huang T, Wang X, Arslan PE (2015). Appoptosin-Mediated Caspase Cleavage of Tau Contributes to Progressive Supranuclear Palsy Pathogenesis. Neuron.

[24] D’Amelio M, Cavallucci V, Middei S, Marchetti C, Pacioni S, Ferri A, Diamantini A, De Zio D, Carrara P, Battistini L (2011). Caspase-3 triggers early synaptic dysfunction in a mouse model of Alzheimer’s disease. Nat Neurosci.

[25] Ding H, Matthews TA, Johnson GV (2006). Site-specific phosphorylation and caspase cleavage differentially impact tau-microtubule interactions and tau aggregation. J Biol Chem.

[26] Fasulo L, Ugolini G, Cattaneo A (2005). Apoptotic effect of caspase-3 cleaved tau in hippocampal neurons and its potentiation by tau FTDP-mutation N279K. J Alzheimers Dis.

[27] Gamblin TC, Chen F, Zambrano A, Abraha A, Lagalwar S, Guillozet AL, Lu M, Fu Y, Garcia-Sierra F, LaPointe N (2003). Caspase cleavage of tau: linking amyloid and neurofibrillary tangles in Alzheimer’s disease. Proc Natl Acad Sci U S A.

[28] Mandelkow E, von Bergen M, Biernat J, Mandelkow EM (2007). Structural principles of tau and the paired helical filaments of Alzheimer’s disease. Brain Pathol.

[29] Rissman RA, Poon WW, Blurton-Jones M, Oddo S, Torp R, Vitek MP, LaFerla FM, Rohn TT, Cotman CW (2004). Caspase-cleavage of tau is an early event in Alzheimer disease tangle pathology. J Clin Invest.

[30] Bright J, Hussain S, Dang V, Wright S, Cooper B, Byun T, Ramos C, Singh A, Parry G, Stagliano N, Griswold-Prenner I (2015). Human secreted tau increases amyloid-beta production. Neurobiol Aging.

[31] Ghochikyan A, Davtyan H, Petrushina I, Hovakimyan A, Movsesyan N, Davtyan A, Kiyatkin A, Cribbs DH, Agadjanyan MG (2013). Refinement of a DNA based Alzheimer’s disease epitope vaccine in rabbits. Hum Vaccin Immunother.

[32] Evans CF, Davtyan H, Petrushina I, Hovakimyan A, Davtyan A, Hannaman D, Cribbs DH, Agadjanyan MG, Ghochikyan A (2014). Epitope-based DNA vaccine for Alzheimer’s disease: Translational study in macaques. Alzheimers Dement.

[33] Davtyan H, Ghochikyan A, Petrushina I, Hovakimyan A, Davtyan A, Cribbs DH, Agadjanyan MG (2014). The MultiTEP platform-based Alzheimer’s disease epitope vaccine activates a broad repertoire of T helper cells in nonhuman primates. Alzheimers Dement.

[34] Davtyan H, Zagorski K, Rajapaksha H, Hovakimyan A, Davtyan A, Petrushina I, Kazarian K, Cribbs DH, Petrovsky N, Agadjanyan MG, Ghochikyan A (2016). Alzheimer’s disease Advax(CpG)- adjuvanted MultiTEP-based dual and single vaccines induce high-titer antibodies against various forms of tau and Abeta pathological molecules. Sci Rep.

[35] Hwang WY, Foote J (2005). Immunogenicity of engineered antibodies. Methods.

[36] Furman JL, Holmes BB, Diamond MI: Sensitive Detection of Proteopathic Seeding Activity with FRET Flow Cytometry. J Vis Exp. 2015;106:e53205. 10.3791/53205 PMC4692784 26710240

[37] Holmes BB, Furman JL, Mahan TE, Yamasaki TR, Mirbaha H, Eades WC, Belaygorod L, Cairns NJ, Holtzman DM, Diamond MI (2014). Proteopathic tau seeding predicts tauopathy *in vivo*. Proc Natl Acad Sci U S A.

[38] Ballatore C, Lee VM, Trojanowski JQ (2007). Tau-mediated neurodegeneration in Alzheimer’s disease and related disorders. Nat Rev Neurosci.

[39] Jack CR, Knopman DS, Jagust WJ, Petersen RC, Weiner MW, Aisen PS, Shaw LM, Vemuri P, Wiste HJ, Weigand SD (2013). Tracking pathophysiological processes in Alzheimer’s disease: an updated hypothetical model of dynamic biomarkers. Lancet Neurol.

[40] Bateman RJ, Xiong C, Benzinger TL, Fagan AM, Goate A, Fox NC, Marcus DS, Cairns NJ, Xie X, Blazey TM (2012). Clinical and biomarker changes in dominantly inherited Alzheimer’s disease. N Engl J Med.

[41] Gerson JE, Kayed R (2013). Formation and propagation of tau oligomeric seeds. Front Neurol.

[42] Clavaguera F, Lavenir I, Falcon B, Frank S, Goedert M, Tolnay M (2013). “Prion-like” templated misfolding in tauopathies. Brain Pathol.

[43] Yanamandra K, Kfoury N, Jiang H, Mahan TE, Ma S, Maloney SE, Wozniak DF, Diamond MI, Holtzman DM (2013). Anti-tau antibodies that block tau aggregate seeding *in vitro* markedly decrease pathology and improve cognition *in vivo*. Neuron.

[44] Frost B, Diamond MI (2010). Prion-like mechanisms in neurodegenerative diseases. Nat Rev Neurosci.

[45] Arrigada PV, Growdon JH, Hedley-White ET, Hyman BT (1992). Neurofibrillary tangles but not senile plaques parallel duration and severity of Alzheimer’s disease. Neurol.

[46] Gomez-Isla T, Hollister R, West H, Mui S, Growdon JH, Petersen RC, Parisi JE, Hyman BT (1997). Neuronal loss correlates with but exceeds neurofibrillary tangles in Alzheimer’s disease. Ann Neurol.

[47] Gu J, Sigurdsson EM (2011). Immunotherapy for tauopathies. J Mol Neurosci.

[48] Himmelstein DS, Ward SM, Lancia JK, Patterson KR, Binder LI (2012). Tau as a therapeutic target in neurodegenerative disease. Pharmacol Ther.

[49] Pedersen JT, Sigurdsson EM (2015). Tau immunotherapy for Alzheimer’s disease. Trends Mol Med.

[50] Braak H, Braak E (1997). Diagnostic criteria for neuropathologic assessment of Alzheimer’s disease. Neurobiol Aging.

[51] Clavaguera F, Bolmont T, Crowther RA, Abramowski D, Frank S, Probst A, Fraser G, Stalder AK, Beibel M, Staufenbiel M (2009). Transmission and spreading of tauopathy in transgenic mouse brain. Nat Cell Biol.

[52] Frost B, Jacks RL, Diamond MI (2009). Propagation of tau misfolding from the outside to the inside of a cell. J Biol Chem.

[53] Iba M, Guo JL, McBride JD, Zhang B, Trojanowski JQ, Lee VM (2013). Synthetic tau fibrils mediate transmission of neurofibrillary tangles in a transgenic mouse model of Alzheimer’s-like tauopathy. J Neurosci.

[54] Lasagna-Reeves CA, Castillo-Carranza DL, Sengupta U, Guerrero-Munoz MJ, Kiritoshi T, Neugebauer V, Jackson GR, Kayed R (2012). Alzheimer brain-derived tau oligomers propagate pathology from endogenous tau. Sci Rep.

[55] Pooler AM, Polydoro M, Wegmann S, Nicholls SB, Spires-Jones TL, Hyman BT (2013). Propagation of tau pathology in Alzheimer’s disease: identification of novel therapeutic targets. Alzheimers Res Ther.

[56] Congdon EE, Gu J, Sait HB, Sigurdsson EM (2013). Antibody uptake into neurons occurs primarily via clathrin-dependent Fcgamma receptor endocytosis and is a prerequisite for acute tau protein clearance. J Biol Chem.

[57] Gu J, Congdon EE, Sigurdsson EM: Two novel tau antibodies targeting the 396/404 region are primarily taken up by neurons and reduce tau pathology. J Biol Chem. 2013;288(46):33081–95. 10.1074/jbc.M113.494922 PMC3829157 24089520

[58] McEwan WA, Falcon B, Vaysburd M, Clift D, Oblak AL, Ghetti B, Goedert M, James LC (2017). Cytosolic Fc receptor TRIM21 inhibits seeded tau aggregation. Proc Natl Acad Sci U S A.

[59] Funk KE, Mirbaha H, Jiang H, Holtzman DM, Diamond MI (2015). Distinct Therapeutic Mechanisms of Tau Antibodies: Promoting Microglial Clearance Versus Blocking Neuronal Uptake. J Biol Chem.

[60] Lee SH, Le Pichon CE, Adolfsson O, Gafner V, Pihlgren M, Lin H, Solanoy H, Brendza R, Ngu H, Foreman O (2016). Antibody-Mediated Targeting of Tau *In Vivo* Does Not Require Effector Function and Microglial Engagement. Cell Rep.

[61] Luo W, Liu W, Hu X, Hanna M, Caravaca A, Paul SM (2015). Microglial internalization and degradation of pathological tau is enhanced by an anti-tau monoclonal antibody. Sci Rep.

[62] Rosenmann H, Grigoriadis N, Karussis D, Boimel M, Touloumi O, Ovadia H, Abramsky O (2006). Tauopathy-like abnormalities and neurologic deficits in mice immunized with neuronal tau protein. Arch Neurol.

[63] Boutajangout A, Ingadottir J, Davies P, Sigurdsson EM (2011). Passive immunization targeting pathological phospho-tau protein in a mouse model reduces functional decline and clears tau aggregates from the brain. J Neurochem.

[64] d’Abramo C, Acker CM, Jimenez HT, Davies P (2013). Tau passive immunotherapy in mutant P301L mice: antibody affinity versus specificity. PLoS One.

[65] d’Abramo C, Acker CM, Jimenez H, Davies P (2015). Passive Immunization in JNPL3 Transgenic Mice Using an Array of Phospho-Tau Specific Antibodies. PLoS One.

[66] Chai X, Wu S, Murray TK, Kinley R, Cella CV, Sims H, Buckner N, Hanmer J, Davies P, O’Neill MJ (2011). Passive immunization with anti-Tau antibodies in two transgenic models: reduction of Tau pathology and delay of disease progression. J Biol Chem.

[67] Sankaranarayanan S, Barten DM, Vana L, Devidze N, Yang L, Cadelina G, Hoque N, DeCarr L, Keenan S, Lin A (2015). Passive immunization with phospho-tau antibodies reduces tau pathology and functional deficits in two distinct mouse tauopathy models. PLoS One.

[68] Collin L, Bohrmann B, Gopfert U, Oroszlan-Szovik K, Ozmen L, Gruninger F (2014). Neuronal uptake of tau/pS422 antibody and reduced progression of tau pathology in a mouse model of Alzheimer’s disease. Brain.

[69] Dai CL, Chen X, Kazim SF, Liu F, Gong CX, Grundke-Iqbal I, Iqbal K (2015). Passive immunization targeting the N-terminal projection domain of tau decreases tau pathology and improves cognition in a transgenic mouse model of Alzheimer disease and tauopathies. J Neural Transm (Vienna).

[70] Ittner LM, Gotz J (2011). Amyloid-beta and tau--a toxic pas de deux in Alzheimer’s disease. Nat Rev Neurosci.

[71] Umeda T, Eguchi H, Kunori Y, Matsumoto Y, Taniguchi T, Mori H, Tomiyama T (2015). Passive immunotherapy of tauopathy targeting pSer413-tau: a pilot study in mice. Ann Clin Transl Neurol.

[72] Yanamandra K, Jiang H, Mahan TE, Maloney SE, Wozniak DF, Diamond MI, Holtzman DM (2015). Anti-tau antibody reduces insoluble tau and decreases brain atrophy. Ann Clin Transl Neurol.

[73] Boutajangout A, Quartermain D, Sigurdsson EM (2010). Immunotherapy targeting pathological tau prevents cognitive decline in a new tangle mouse model. J Neurosci.

[74] Boimel M, Grigoriadis N, Lourbopoulos A, Haber E, Abramsky O, Rosenmann H (2010). Efficacy and safety of immunization with phosphorylated tau against neurofibrillary tangles in mice. Exp Neurol.

[75] Bi M, Ittner A, Ke YD, Gotz J, Ittner LM (2011). Tau-targeted immunization impedes progression of neurofibrillary histopathology in aged P301L tau transgenic mice. PLoS One.

[76] Rasool S, Martinez-Coria H, Wu JW, LaFerla F, Glabe CG (2013). Systemic vaccination with anti-oligomeric monoclonal antibodies improves cognitive function by reducing Abeta deposition and tau pathology in 3xTg-AD mice. J Neurochem.

[77] Asuni AA, Boutajangout A, Quartermain D, Sigurdsson EM (2007). Immunotherapy targeting pathological tau conformers in a tangle mouse model reduces brain pathology with associated functional improvements. J Neurosci.

[78] Troquier L, Caillierez R, Burnouf S, Fernandez-Gomez FJ, Grosjean ME, Zommer N, Sergeant N, Schraen-Maschke S, Blum D, Buee L (2012). Targeting phospho-Ser422 by active Tau Immunotherapy in the THYTau22 mouse model: a suitable therapeutic approach. Curr Alzheimer Res.

[79] Theunis C, Crespo-Biel N, Gafner V, Pihlgren M, Lopez-Deber MP, Reis P, Hickman DT, Adolfsson O, Chuard N, Ndao DM (2013). Efficacy and safety of a liposome-based vaccine against protein Tau, assessed in tau.P301L mice that model tauopathy. PLoS One.

[80] Zilka N, Filipcik P, Koson P, Fialova L, Skrabana R, Zilkova M, Rolkova G, Kontsekova E, Novak M (2006). Truncated tau from sporadic Alzheimer’s disease suffices to drive neurofibrillary degeneration *in vivo*. FEBS Lett.

[81] Kontsekova E, Zilka N, Kovacech B, Novak P, Novak M (2014). First-in-man tau vaccine targeting structural determinants essential for pathological tau-tau interaction reduces tau oligomerisation and neurofibrillary degeneration in an Alzheimer’s disease model. Alzheimers Res Ther.

[82] Neuber T, Frese K, Jaehrling J, Jager S, Daubert D, Felderer K, Linnemann M, Hohne A, Kaden S, Kolln J (2014). Characterization and screening of IgG binding to the neonatal Fc receptor. MAbs.

[83] Gurbaxani B, Dela Cruz LL, Chintalacharuvu K, Morrison SL (2006). Analysis of a family of antibodies with different half-lives in mice fails to find a correlation between affinity for FcRn and serum half-life. Mol Immunol.

[84] Dai CL, Tung YC, Liu F, Gong CX, Iqbal K (2017). Tau passive immunization inhibits not only tau but also Abeta pathology. Alzheimers Res Ther.

[85] Castillo-Carranza DL, Gerson JE, Sengupta U, Guerrero-Munoz MJ, Lasagna-Reeves CA, Kayed R (2014). Specific targeting of tau oligomers in Htau mice prevents cognitive impairment and tau toxicity following injection with brain-derived tau oligomeric seeds. J Alzheimers Dis.

[86] Kfoury N, Holmes BB, Jiang H, Holtzman DM, Diamond MI (2012). Trans-cellular propagation of Tau aggregation by fibrillar species. J Biol Chem.

[87] Levites Y, Smithson LA, Price RW, Dakin RS, Yuan B, Sierks MR, Kim J, McGowan E, Reed DK, Rosenberry TL (2006). Insights into the mechanisms of action of anti-Abeta antibodies in Alzheimer’s disease mouse models. FASEB J.

[88] Atwal JK, Chen Y, Chiu C, Mortensen DL, Meilandt WJ, Liu Y, Heise CE, Hoyte K, Luk W, Lu Y (2011). A therapeutic antibody targeting BACE1 inhibits amyloid-beta production *in vivo*. Sci Transl Med.

[89] Yu YJ, Zhang Y, Kenrick M, Hoyte K, Luk W, Lu Y, Atwal J, Elliott JM, Prabhu S, Watts RJ, Dennis MS (2011). Boosting brain uptake of a therapeutic antibody by reducing its affinity for a transcytosis target. Sci Transl Med.

[90] Xiao C, Davis FJ, Chauhan BC, Viola KL, Lacor PN, Velasco PT, Klein WL, Chauhan NB (2013). Brain transit and ameliorative effects of intranasally delivered anti-amyloid-beta oligomer antibody in 5XFAD mice. J Alzheimers Dis.

[91] Chauhan MB, Chauhan NB: Brain Uptake of Neurotherapeutics after Intranasal versus Intraperitoneal Delivery in Mice. J Neurol Neurosurg. 2015;2(1). PMC4567259 26366437

[92] Davtyan H, Ghochikyan A, Petrushina I, Hovakimyan A, Davtyan A, Poghosyan A, Marleau AM, Movsesyan N, Kiyatkin A, Rasool S (2013). Immunogenicity, Efficacy, Safety, and Mechanism of Action of Epitope Vaccine (Lu AF20513) for Alzheimer’s Disease: Prelude to a Clinical Trial. J Neurosci.

[93] Lasagna-Reeves CA, Castillo-Carranza DL, Guerrero-Muoz MJ, Jackson GR, Kayed R (2010). Preparation and characterization of neurotoxic tau oligomers. Biochemistry.

[94] Davtyan H, Mkrtichyan M, Movsesyan N, Petrushina I, Mamikonyan G, Cribbs DH, Agadjanyan MG, Ghochikyan A (2010). DNA prime-protein boost increased the titer, avidity and persistence of anti-Abeta antibodies in wild-type mice. Gene Ther.

